# High biocompatible FITC-conjugated silica nanoparticles for cell labeling in both in vitro and in vivo models

**DOI:** 10.1038/s41598-024-55600-w

**Published:** 2024-03-23

**Authors:** Thi Thuy Nguyen, Hoang Nam Nguyen, Thi Ha Lien Nghiem, Xuan-Hai Do, Thanh Thuy To, Thi Xuan Phuong Do, Dieu Linh Do, Huong Giang Nguyen, Huy Manh Nguyen, Ngoc Dinh Nguyen, Manh Quynh Luu, Trong Nghia Nguyen, Thi Bich Ngoc Nguyen, Van Toan Nguyen, Van Thanh Pham, Uyen Thi Trang Than, Thi My Nhung Hoang

**Affiliations:** 1 Center for Quantum and Electronics, Institute of Physics, Vietnam Academy of Science and Technology, 18 Hoang Quoc Viet Street, Hanoi, Vietnam; 2 Nano and Energy Center, VNU University of Science, Hanoi, 334 Nguyen Trai Street, Thanh Xuan, Hanoi, Vietnam; 3 Department of Practical and Experimental Surgery, Vietnam Military Medical University, 160 Phung Hung Street, Phuc La, Ha Dong, Hanoi, Vietnam; 4 Faculty of Biology, VNU University of Science, Hanoi, 334 Nguyen Trai Street, Thanh Xuan, Hanoi, 10000 Vietnam; 5 Faculty of Physics, VNU University of Science, Hanoi, 334 Nguyen Trai Street, Thanh Xuan, Hanoi, Vietnam; 6 Vinmec Hi-Tech Center and Vinmec-VinUni Institute of Immunology, Vinmec Healthcare System, 458 Minh Khai Street, Hanoi, Vietnam

**Keywords:** Silica nanoparticles, Fluorescein isothiocyanate, Biocompatibility, Cell labelling, Nanobiotechnology, Stem cells

## Abstract

Fluorescence nanosilica-based cell tracker has been explored and applied in cell biological research. However, the aggregation of these nanoparticles at physiological pH is still the main limitation. In this research, we introduced a novel fluorescence nano-based cell tracker suitable for application in live cells. The silica-coated fluorescein isothiocyanate isomer (FITC-SiO_2_) nanoparticles (NPs) were modified with carboxymethylsilanetriol disodium salt (FITC-SiO_2_-COOH), integrating the dianion form of FITC molecules. This nanosystem exhibited superior dispersion in aqueous solutions and effectively mitigated dye leakage. These labeled NPs displayed notable biocompatibility and minimal cytotoxicity in both in vitro and in vivo conditions. Significantly, the NPs did not have negative implications on cell migration or angiogenesis. They successfully penetrated primary fibroblasts, human umbilical vein endothelial cells and HeLa cells in both 2D and 3D cultures, with the fluorescence signal enduring for over 72 h. Furthermore, the NP signals were consistently observed in the developing gastrointestinal tract of live medaka fish larvae for extended periods during phases of subdued digestive activity, without manifesting any apparent acute toxicity. These results underscore the promising utility of FITC-SiO_2_-COOH NPs as advanced live cell trackers in biological research.

## Introduction

Fluorescein isothiocyanate isomer (FITC) is a prominent imaging modality in biomedical sciences for visualizing cells and tissues both in vitro and in vivo^1–4^. The primary advantages of this dye include high contrast visualization, elevated sensitivity, affordability, and ease of manipulation, which render it an invaluable tool for research. Nevertheless, notable drawbacks associated with this fluorescein for biomedical applications include potential cytotoxicity, small scale susceptibility to photobleaching, and diminished fluorescence signal intensities. Additionally, its limited colloidal stability under physiological conditions hinders its broader biological application. Incorporating these organic dyes into polymers or inorganic silica SiO_2_ matrices can mitigate such issues^5–12^. Particularly, SiO_2_ stands out as an optimal material for biomedical applications due to its distinctive properties, such as chemical stability, optical transparency, biocompatibility, and amenability to functionalization. Encapsulating FITC molecules within the SiO_2_ matrix offers enhanced and sustained fluorescence^7^. This encapsulation method also stabilizes FITC against pH-related variations and shields it from photobleaching^13,14^.

Moreover, the SiO_2_ nanoparticle (NP) surface can be adeptly modified by grafting organosilanes, phosphonic acids, DIO or carboxylate-based ligands, or polymers bearing amino terminal groups, depending on the desired surface modification^11,15–17^. Consequently, FITC-SiO_2_ nanoparticles (NPs) exhibit superior colloidal stability in solutions and feature functional groups that offer a platform for immobilizing various biomolecules, from small chemical entities to DNA, proteins, and nucleotides. These attributes earmark them as promising nanocarriers for applications spanning cell imaging, photodynamic therapy, diagnostics, bioanalysis, and the delivery of drugs and bioactive agents, such as proteins and genes.

Various methodologies have been developed to produce FITC-SiO_2_ NPs^14,18^. A prevalent approach involves trapping the dyes within the particles, usually initiated by a covalent pre-conjugation of the dye to a suitable functional group. Efficient entities for this covalent FITC trapping include the amino group of (3-Aminopropyl) triethoxysilane (APTES)^19^ and chitosan^1^. This covalent bond ensures that FITC molecules are securely encapsulated within the silica matrix, shielded from environmental agents. Yet, the amino groups on the silica surface can become protonated (-NH3^+^) and acquire a positive charge at physiological pH. This alteration can reduce the overall surface charge, promoting particle aggregation. Hence, additional silica surface modifications, such as incorporating carboxylic and thiol groups, have been suggested to enhance stability in both in vivo and in vitro contexts^17,18^.

In this study, we present, for the first time, the synthesis and characterization of a novel labeling nanoparticle system: FITC-SiO_2_ functionalized by carboxyl groups derived from carboxymethylsilanetriol disodium salt, denoted as FITC-SiO_2_-COOH. This nanosystem is notably stable in aqueous solutions, resistant to dye leakage, exhibits commendable biocompatibility, and has minimal cytotoxicity. Moreover, it displayed no detrimental effects on cell migration or in vitro angiogenesis. Significantly, FITC-SiO_2_-COOH NPs could penetrate primary fibroblasts (hFBs), human umbilical vein endothelial cells (hUVECs), and cervical cancer cell line (HeLa) in both 2D and 3D cultures, maintaining fluorescence for over 72 h. This suggests the potential of FITC-SiO_2_-COOH NPs as a proficient live cell monitoring.

## Results

### Synthesize and characterization of FITC-SiO_2_-COOH nanoparticles

**Figure 1:**
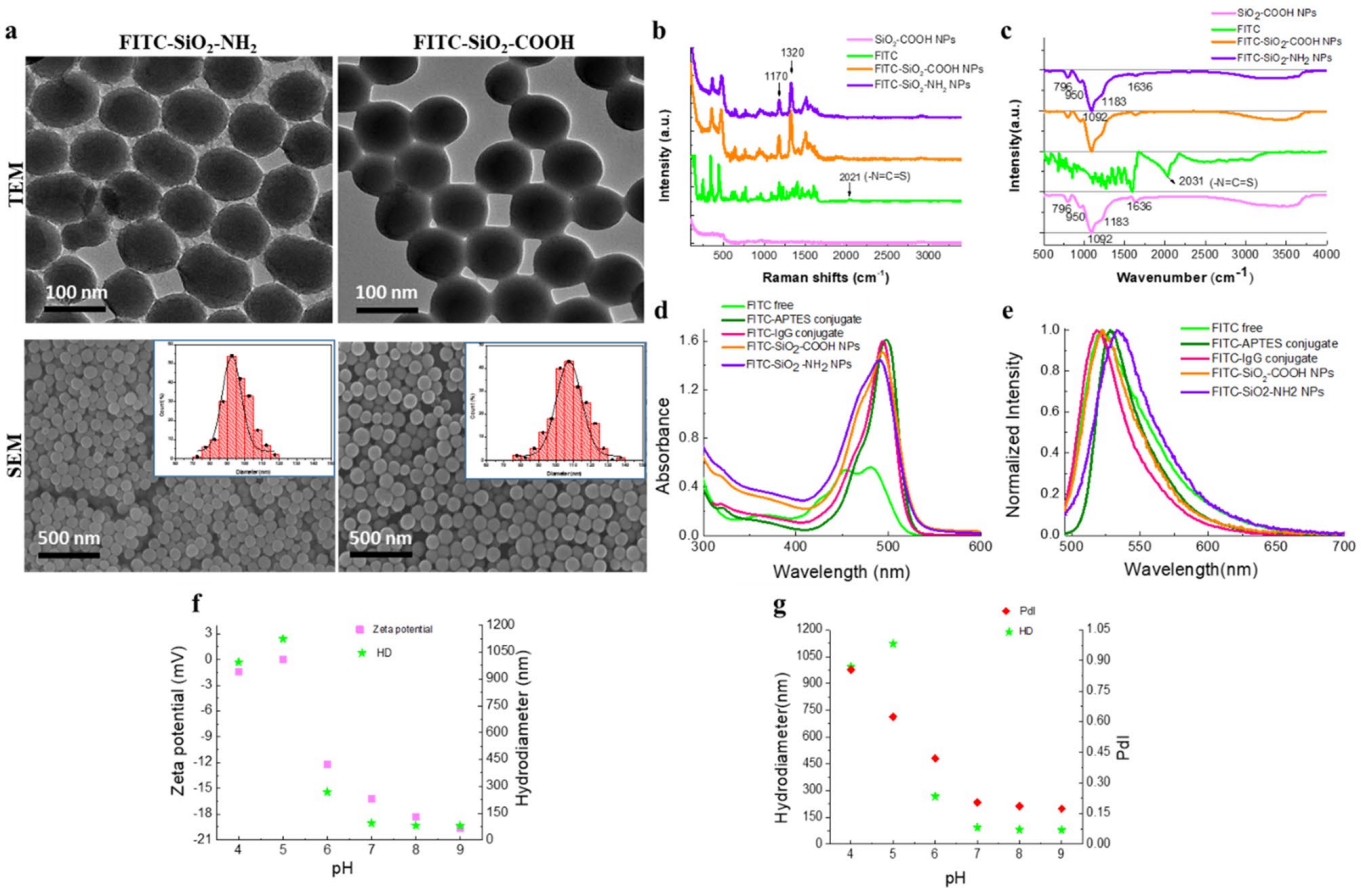
Nanoparticle characterization. (**a**) TEM and SEM images depicting FITC-SiO_2_-NH_2_ and FITC-SiO_2_-COOH NPs. (**b**) Raman and (**c**) FTIR spectra of SiO_2_-COOH NPs (magenta line), FITC (green line), FITC-SiO_2_-COOH NPs (orange line), and FITC-SiO_2_-NH_2_ NPs (violet line). UV–Vis spectra (**d**) and their corresponding normalized fluorescent spectra (excitation 480 nm) (**e**) of free FITC (green line), FITC conjugated with APTES in ethanol (olive line), FITC-conjugated IgG (magenta line) and FITC-SiO_2_-COOH NPs (orange line), and FITC-SiO_2_-NH_2_ NPs (violet line) in water. Zeta potential values (**f**) and poly index dispersion values (**g**) of FITC-SiO_2_-COOH NPs at different pH values. *HD* hydrodynamic diameter.

The morphology and structure of FITC-SiO_2_-NH_2_ and FITC-SiO_2_-COOH NPs were visualized using transmission electron microscopy (TEM) and scanning electron microscopy (SEM) as depicted in Fig. 1a. Both types of nanoparticles predominantly exhibited a spherical shape, a smooth surface, and a consistent size. The inset images in Fig. 1a are the size distribution of FITC-SiO_2_-NH_2_ NPs and FITC-SiO_2_-COOH NPs from corresponding TEM and SEM images. The diameter of the particles was determined using the open-source Image-J software. From there, it is possible to decide on the nanoparticles' average size and corresponding standard deviation. Specifically, the average diameters for FITC-SiO_2_-NH_2_ and FITC-SiO_2_-COOH NPs were 93 ± 12 nm and 107 ± 17 nm, respectively. Dynamic Light Scattering (DLS) and Zeta potentiometry analyses, presented in Table 1, were utilized to assess their colloidal stability in water. The DLS results revealed a monomodal hydrodynamic size distribution with relatively low polydispersity index (PdI) values. The hydrodynamic diameter of FITC-SiO_2_-COOH NPs was registered at 115 nm, a slight increment from the FITC-SiO_2_-NH_2_ NPs which ranged between 109 to 115 nm. Moreover, the measured Zeta potentials were − 11 mV for FITC-SiO_2_-NH_2_ and − 42.4 mV for FITC-SiO_2_-COOH. This shift towards a more negative value for the latter can be attributed to the presence of hydroxyl and amino groups on the surface of FITC-SiO_2_-NH_2_ NPs. These groups result in a minor negative zeta potential and consequentially higher aggregation tendencies. The significantly negative zeta potential of FITC-SiO_2_-COOH NPs (− 42.4 mV) is indicative of carboxylate COO^−^ groups present on their surface, suggesting that they remain highly dispersed^7^.

**Table 1:** Characterization data of FITC-SiO_2_-NH_2_ and FITC-SiO_2_-COOH NPs: average particle size determined by TEM (d_TEM_), standard deviation (σ_TEM_), average hydrodynamic diameter inferred from DLS (d_DLS_), PdI, and zeta potential.

Sample	d_TEM_ (nm)	σ_TEM_ (nm)	d_DLS_ (nm)	PdI	Zeta potential (mV)
FITC-SiO_2_-NH_2_	93	12	109	0.024	− 11.2
FITC-SiO_2_-COOH	107	17	115	0.017	− 42.4

For Raman and Fourier Transform Infrared Spectroscopy (FTIR) measurements, SiO_2_-COOH and FITC-SiO_2_-COOH NPs were centrifugally separated and subsequently dried to produce anhydrous powders. The Raman and FTIR spectra of SiO_2_-COOH NPs, FITC-SiO_2_-COOH NPs, and pure FITC molecules are presented in Fig. 1b, c. Each Raman spectrum was captured under uniform conditions as following: 60 s duration, 785 nm laser excitation, 600 line/mm grating, and utilizing a 100× optical microscope objective. The findings indicate pronounced Raman effects (Fig. 1b). The Raman signal of SiO_2_-COOH NPs (magenta line) was weak compared to those of FITC-SiO_2_-COOH NPs (orange line), FITC-SiO_2_-NH_2_ NPs (violet line), and the FITC powder sample (green line, divided by 20, shown in Fig. 1c), which indicated that the Raman activity of SiO_2_-COOH NPs was considerably muted. In contrast, the Raman signature of FITC-SiO_2_-COOH and FITC-SiO_2_-NH_2_ NPs were robust and echoed characteristic peaks of the FITC molecules (orange line). Notably, the peaks at 1170 cm^−1^ and 1320 cm^−1^ are ascribed to the CCH bend of the xanthene ring and the C-O (phenoxide ion stretch conjugated with xanthene ring stretch, respectively), which is a characteristic of the dianion forms of FITC molecules and became more pronounced^8^. The disappearance of the peak at 2021 cm^−1^ assigned to the –N=C=S groups proved the covalent bonding of FITC conjugated with the silica matrix via the APTES molecule^9^.

The pure FITC molecule exhibited distinct spectra in both Raman and FTIR readings (Fig. 1c). In the FTIR spectrum of FITC molecules (green line), a pronounced peak at 2031 cm^−1^ corresponded to the stretch vibration of the –N=C=S group. Following the covalent attachment of FITC to the silica nanoparticle network, this peak was no longer discernible in the FTIR spectrum of the FITC-SiO_2_-COOH NPs (orange line) and FITC-SiO_2_-NH_2_ (violet line), affirming the conjugation of FITC with the silica matrix via the APTES molecule. Unlike the Raman data, the FTIR spectra for both FITC-SiO_2_-COOH NPs and SiO_2_-COOH NPs closely resembled the standard silica gel FTIR spectrum^9^. There was no discernible peak associated with FITC molecules in the FITC-SiO_2_-COOH and FITC-SiO_2_-NH_2_ NP's FTIR spectrums. Such findings underscore the enhanced sensitivity of the dianion structure of the FITC molecule in the Raman spectrum compared to the infrared spectrum.

The absorption and fluorescence spectra of free FITC, FITC conjugated with APTES in ethanol, FITC-SiO_2_-COOH NPs, and antibody-FITC in water are presented in Fig. 1d, e. Figure 1d depicts the UV–Vis spectrum of free FITC in ethanol, representative of the electronic absorption spectrum of the anion form of FITC molecules^8^. The absorption spectra of FITC conjugated with APTES in ethanol showed a peak at 498 nm (olive line), while FITC conjugated with the IgG antibody in water peaked at 494 nm (magenta line). Notably, conjugated FITC samples exhibited narrow absorption, resembling those of the dianion forms of FITC at high pH. The FITC-SiO_2_-COOH NPs (orange line) and FITC-SiO_2_-NH_2_ NPs (violet line) in water revealed a broad absorption spectrum with a slight blue- shift, peaking at 494 nm and 492 nm corresponding, comparable to the FITC conjugated with IgG antibody. Pure silica exhibited no absorption in its monolithic form; however, as nanoparticles, it enhanced the background spectrum. The variation in absorption spectra is attributable to the xanthene segment of the FITC molecule, which possesses three protonation sites, resulting in multiple forms (cationic, neutral, anionic, and dianionic) that are pH-dependent. Specifically, the fluorescence quantum yield increases from 0.37 in the monoanion form to 0.93 in the dianion form. In Fig. 1e, with an excitation wavelength of 480 nm, typical emission peaks of all samples were identified around 522 nm for both the free FITC molecule and the FITC-SiO_2_-COOH NPs, at 527 nm for the FITC-APTES conjugate, at 532 nm for the FITC-SiO_2_-NH_2_ NPs, and 518 nm for the FITC-IgG conjugate. The fluorescence spectrum of FITC-SiO_2_-COOH NPs resembled that of FITC conjugated with IgG antibodies. These results suggested that, after conjugation with the amino groups of APTES or antibodies, the fluorescence spectral profiles of FITC molecules become more refined.

The system's colloidal stability of FITC-SiO2-COOH NPs was determined by examining the zeta potential, the hydrodynamic diameter of nanoparticles, and their polydispersity index values. The obtained results were presented in Fig. 1f, g. The zeta potential values of the NPs in the buffer systems increased compared to that of the nanoparticles dispersed in deionized water. In these high salty concentration buffers, the results showed that the zeta potential was found to be about 0 at pH 5, − 1.4 mV at pH 4, − 12 mV at pH 6, − 16.2 mV at pH 7, and stable in pH 8, pH 9 with zeta potentials of − 18.3 mV and − 19.6 mV, respectively. By following the variation of the zeta potential of NPs as a function of the pH, the isoelectric point was identified at pH 5. At pH 5, pH 4, and pH 6, the hydrodynamic diameter of the particle was enormous, up to 1200 nm, much larger than the size measured by the TEM, indicating that the particle system was aggregated in this pH range. The farther away from the isoelectric point, in the pH range of 7–9, the nanoparticle system became more stable, and the hydrodynamic size of the particles was relatively similar and close to the diameter obtained from TEM images. This result demonstrated that the FITC-SiO_2_-COOH nanoparticle colloidal system was relatively stable in the pH range greater than 6 and formed aggregates in the low pH range of 4–6. At the same time, it showed that the chemical resistance of this nanoparticle system was excellent. The storage durability of these particles is very long, up to years if stored in deionized water.

### The well distribution and selective penetration of FITC-SiO_2_-COOH NPs in different cell types

In this study, we used two primary cell types, including hFBs and hUVECs, and one cervical cancer cell line HeLa. Using different cell types would inform us if our nanoparticles could be widely applied as living cell trackers in biomedical research. Besides, primary cells, such as hUVECs and hFBS, are derived from tissue and not modified. Thus, these cells could provide a suitable model for studying the normal physiology of the cell response to the tested compounds.

**Figure 2:**
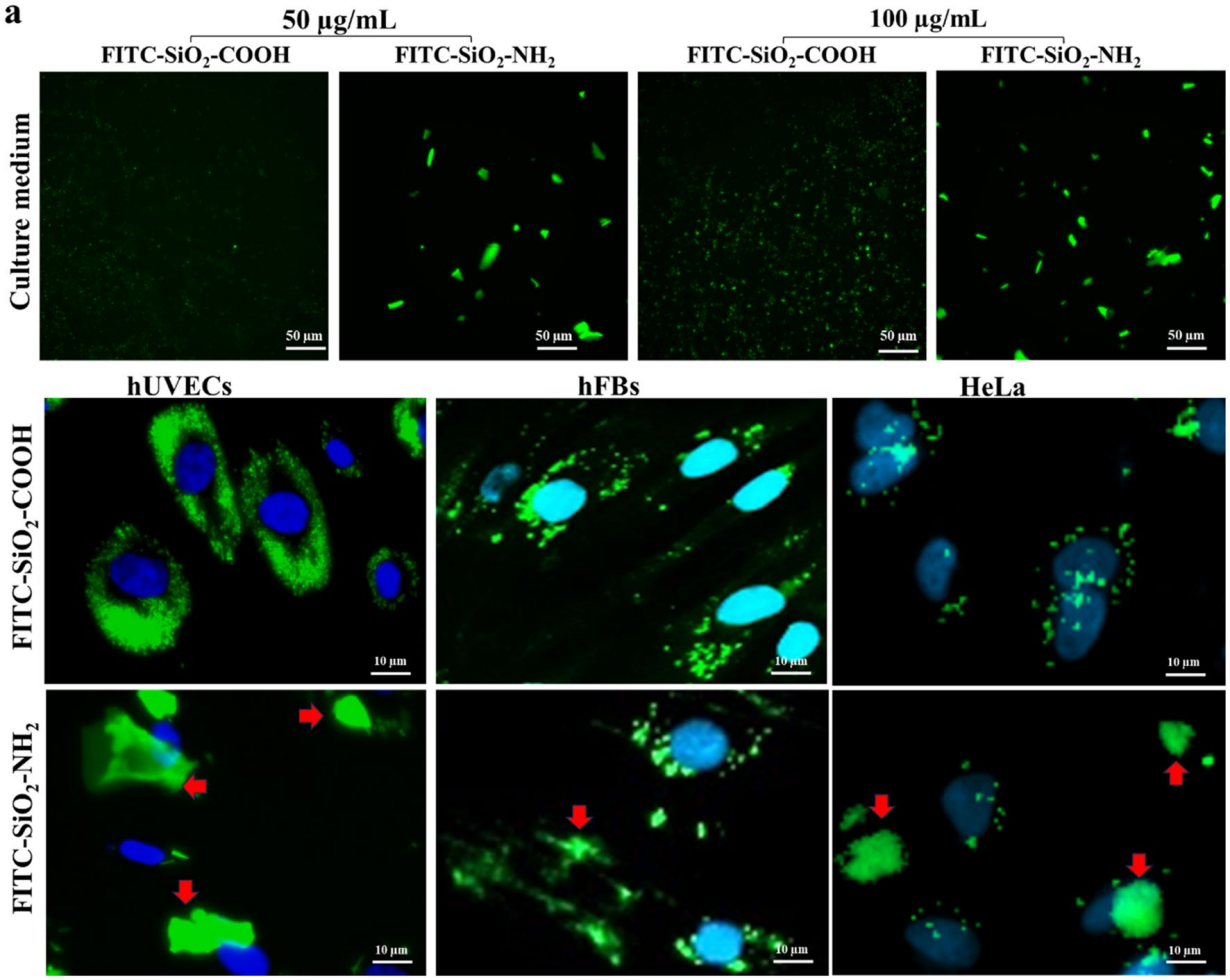
The cell penetration of FITC-SiO2-COOH NPs in different cell types. (**a**) The dispersion of FITC-SiO_2_-COOH and FITC-SiO_2_-NH_2_ NPs at two doses after 30 min of incubation with the cell culture medium. (**b**) The different distributions of two NP types in hUVECs, hFBs, and HeLa cells. The red arrows indicated the localization of NPs outside the cells.

Before adding on the cells, we tested the distribution of the NPs in the cell culture medium. After 30 min, at the two tested doses 50 and 100 µg/mL, the FITC-SiO_2_-NH_2_ NPs gradually aggregated as large as clumps with the average size even bigger than the cell nuclei. In the meantime, FITC-SiO_2_-COOH NPs were well dispersed in the cell culture medium (Fig. 2a). We then incubated these NPs at 100 µg/mL for 24 h with different cell types. Similar results were obtained, that FITC-SiO_2_-NH_2_ NPs were aggregated in all cell culture, consequently, stay outside of the cells (red arrows), even though some of them successfully penetrated to the cytoplasm. Interestingly, FITC-SiO_2_-COOH NPs stayed mainly inside the cells surrounding the cell nuclei, especially in hUVECs and hFBs, and less in HeLa cells (Fig. 2b). These results demonstrated that FITC-SiO_2_-COOH NPs were more effective in cell penetration compared to that of FITC-SiO_2_-NH_2_ NPs in hUVECs, hFBs, and HeLa cells. Therefore, we chose FITC-SiO_2_-COOH NPs for the next biocompatibility evaluations as a cell labeling in hUVECs, hFBs, and HeLa cells.

### The effect of FITC-SiO_2_-COOH NPs on the cytotoxicity and cell senescence

**Figure 3:**
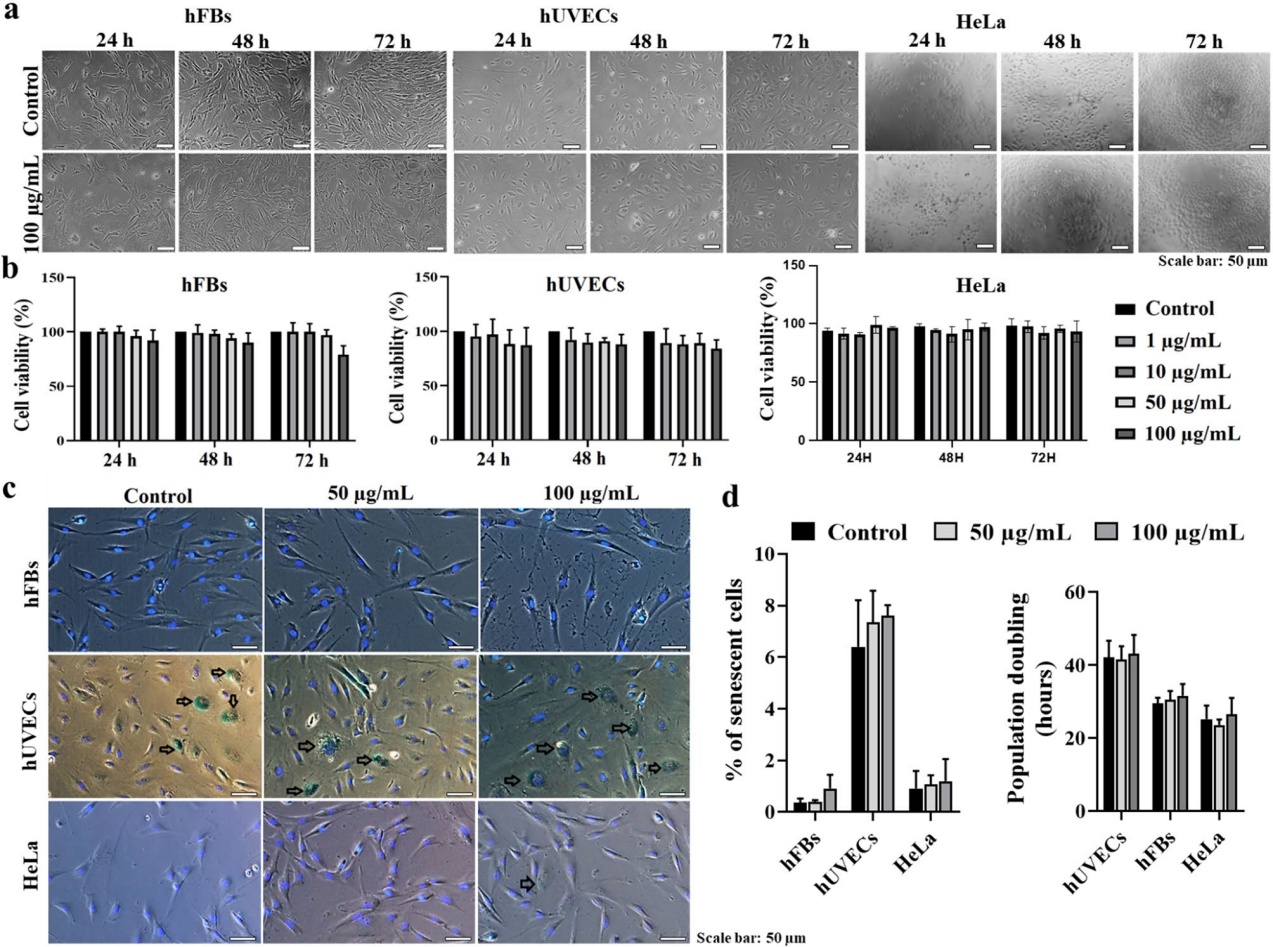
The effect of FTIC-SiO_2_-COOH NPs on cytotoxicity and cell senescence. (**a**) The morphology and cell density of hFBs, hUVECs, and HeLa cells in the presence of the NPs. (**b**) The cell viability (%) of hFBs, hUVECs, and HeLa cells with different doses assessed at 24, 48, and 72 h. (**c**) The cellular senescence signals detected by β-galactosidase staining; black arrows indicate aging cells. Cell nuclei were stained with Hoechst (blue). (**d**) The percentage of senescence cells (%) and the population doubling time among hFBs, hUVECs, and HeLa cells. Data was collected from three biological trials (n = 3) and presented as mean ± SD.

Cell viability was evaluated at 24 h, 48 h, and 72 h post-incubation with the NPs. No significant disparities in cell morphology or density were observed between the three cell types (Fig. 3a). Crucially, both the treated and control cells exhibited no significant deviation in viable cell percentages (Fig. 3b). Additionally, cellular senescence markers in the presence of FITC-SiO_2_-COOH NPs were identified (Fig. 3c). It was evident that hUVECs had a notably higher rate of aging cells (6.4 ± 1.8%) compared to hFBs (0.4 ± 0.15%) and HeLa cells (0.9 ± 0.7%) (Fig. 3d). Yet, the NPs did not expedite the aging process, as similar cellular senescence markers were evident in both the treated and control groups. We also checked the effect of these nanoparticles on the cell population doubling times. The results showed that there was not significant difference in the cell duplication between the control and the treatment at 50 µg/mL and 100 µg/mL NPs in all three cell types (Fig. 3d).

These data underscores that FITC-SiO_2_-COOH NPs did not influence cell viability or aging in either hFBs, hUVECs, and HeLa cells.

### The effect of FITC-SiO_2_-COOH NPs on cell migration and in vitro angiogenesis

**Figure 4:**
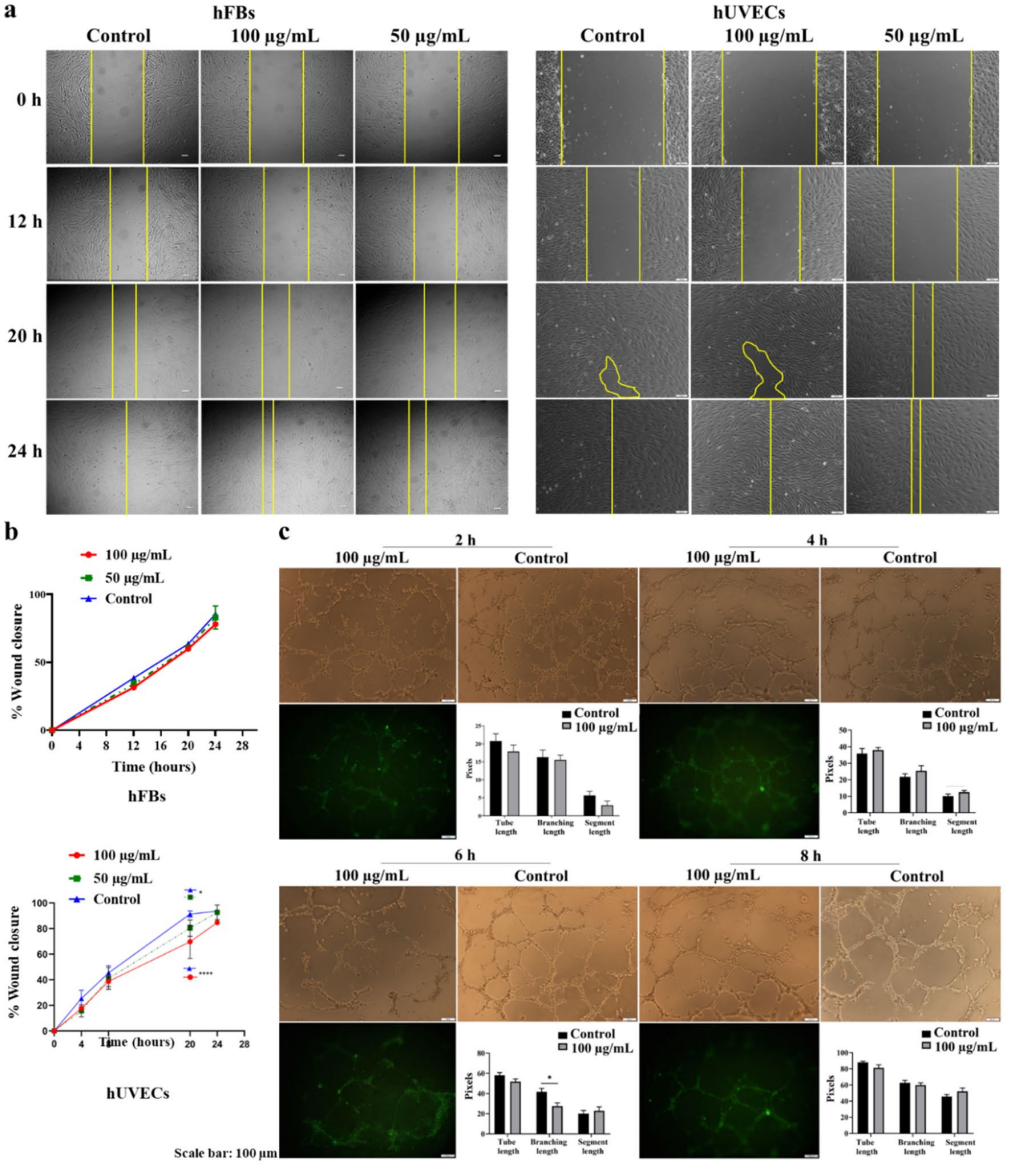
The effect of FTIC-SiO_2_-COOH NPs on cell migration and in vitro angiogenesis. (**a**) The images of wounded sites on the cell monolayers at 0, 12, 20 and 24 h. (**b**) The proportion of wound closure in the presence of NPs at different concentrations with time. (**c**) The effect of FITC-SiO_2_-COOH NPs at 100 µg/mL on in vitro angiogenesis of hUVECs. Data was collected from three biological trials (n = 3) and presented as mean ± SD. *p < 0.05; ****p < 0.0001.

To discern the nanoparticles' influence on cell functionality, a wound healing assay was conducted using FITC-SiO_2_-COOH NPs at concentrations of 50 and 100 µg/mL. Results indicated that the NPs did not hinder the migration of hFBs or hUVECs, irrespective of the concentration (Fig. 4a). Moreover, the wound closure rate was consistent across all assessment times (Fig. 4b). At the elevated concentration of 100 µg/mL, a delay in cell migration was observed compared to controls in hUVECs (*p* < 0.0001) at 20 h, but this delay was not statistically significant (*p* > 0.05) at 24 h (Fig. 4b), suggesting that the NPs did not impede cellular migration in 2D cultures.

Further analysis showed that the NPs had no discernible impact on the tube formation ability of hUVECs. Even at the higher concentration of 100 µg/mL of FITC-SiO_2_-COOH NPs, metrics such as total tube length, branching length, and segment length remained consistent with controls across time periods of 2, 4, and 8 h post-seeding on Matrigel, though there was a minor reduction in branching length in treated cells at 6 h (*p* < 0.05) (Fig. 4c). Intriguingly, the NPs exhibited cell-labeling activity that persisted beyond 8 h of observation, suggesting potential utility as a labeling agent for cell tracking.

### FITC-SiO_2_-COOH NPs function as a cell labeling agent in 2D culture

**Figure 5:**
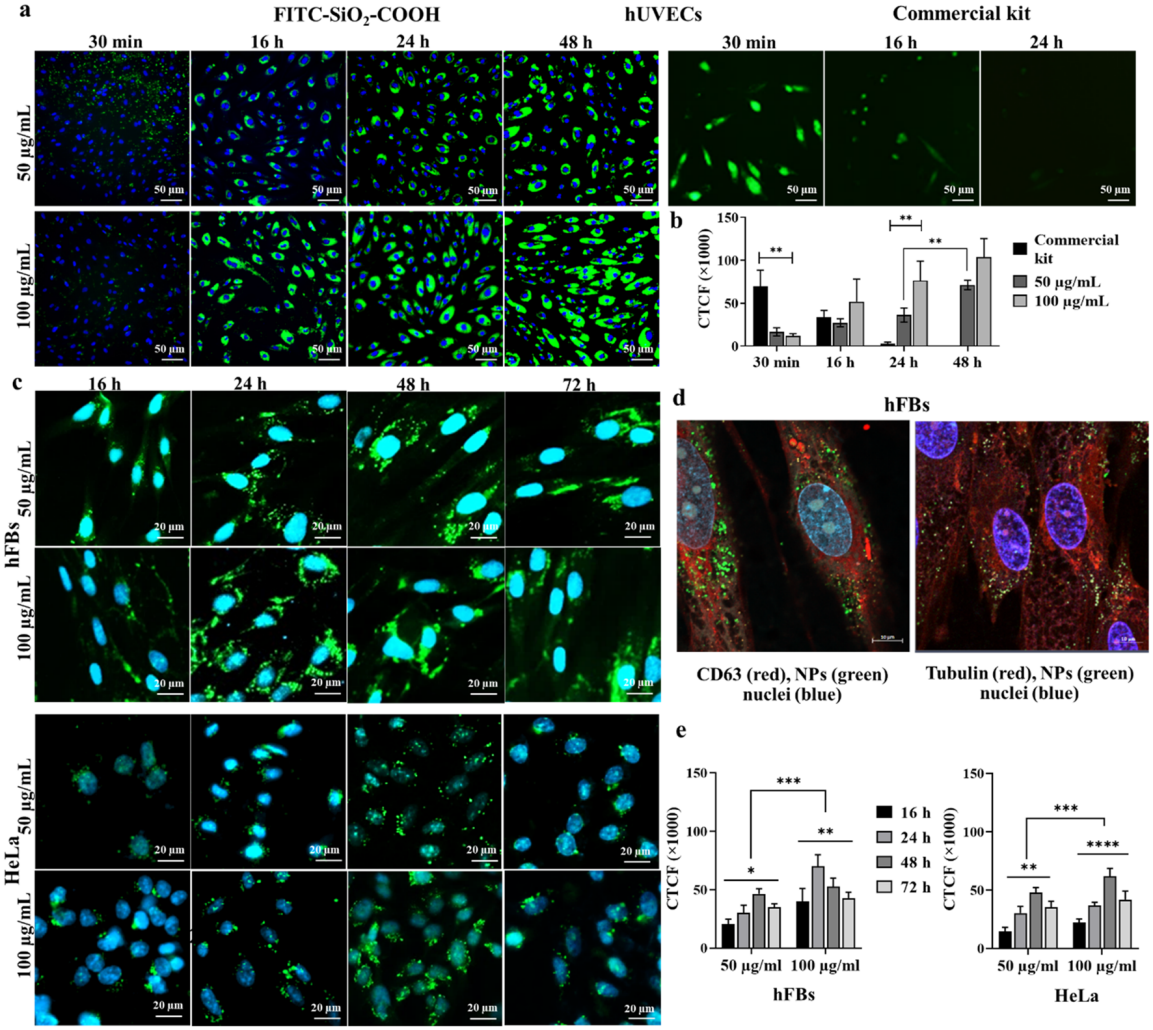
Analysis of fluorescence signals of FITC-SiO_2_-COOH NPs. (**a**) The uptake of FITC-SiO_2_-COOH NPs and a commercial fluorescence kit in the hUVECs after different times of incubation. (**b**) The quantitative comparison of fluorescence signals at different time points of hUVECs and a commercial kit. (**c**) hFBs and HeLa cells incubated with FITC-SiO_2_-COOH NPs at various times. (**d**) The intracellular distribution of FITC-SiO_2_-COOH NPs in hFBs. The relative localization of FITC-SiO_2_-COOH NPs (green) in the cytoplasm and CD63 membrane protein (red, left), or alpha-tubulin (red, right). (**e**) Quantitative comparison of fluorescence signals at different time points at 50 µg/mL and 100 µg/mL NPs in hFBs and HeLa cells. Data was collected from three biological trials (n = 3) and presented as mean ± SD. *p < 0.05; **p < 0.01; ***p < 0.001.

Based on the in vitro angiogenesis assay results, the potential of FITC-SiO_2_-COOH NPs was assessed for their efficacy as a fluorescence labeling agent in live cell tracking. Two concentrations of the NPs were examined. Notably, in hUVECs, following a 24-h incubation, fluorescence was discernible in nearly all cells. The NPs appeared to localize predominantly in the cytoplasm, surrounding the cell nuclei (Fig. 5a). We also compared the fluorescence signal of our NPs with the commercial fluorescence labeling agent provided with the angiogenesis kit at different times of incubation. Remarkably, these NPs maintained their fluorescence within the cells for a longer duration than the commercial dye. As shown in Fig. 5b, hUVECs pre-labeled with the commercial kit exhibited a robust signal after a 30-min incubation, but this rapidly diminished after 16 h, and vanished by the 24-h mark. In contrast, the fluorescence from FITC-SiO_2_-COOH NPs persisted up to 48 h post-incubation. These findings suggest that these NPs could serve as effective long-term live cell labeling agents for hUVECs.

A similar intracellular distribution of FITC-SiO_2_-COOH NPs was observed in fibroblasts and HeLa cells, with the fluorescence signal observed from 16 h and last for 72 h after incubation. The signal was more pronounced in cells treated with 100 µg/mL NPs compared to those treated with 50 µg/mL NPs across all assessment periods (Fig. 5c). To validate the cellular uptake of these NPs, cells were stained with CD63 antibodies to identify cell membrane proteins and alpha-tubulin antibodies to delineate cytoskeletal components. Observations revealed a uniform distribution of NPs within fibroblasts after 24 h of incubation (Fig. 5d).

In hFBs, the peak fluorescence intensity was achieved after 48 h of incubation with the lower dose of NPs, and 24 h with the higher dose. In HeLa cells, the peak fluorescence intensity was achieved after 48 h of incubation with both doses of NPs (Fig. 5e). The signal was still observed after 72 h with the fluorescence intensity decreased of about 45.3%. The fluorescence exhibited a noticeable decline by 72 h, especially for the higher dose in both cell types. Such findings affirm that the NPs successfully penetrated fibroblasts and HeLa cells, serving as cellular trackers for durations exceeding 48 h.

### FITC-SiO_2_-COOH NPs function as a cell labeling agent in 3D cultures

**Figure 6:**
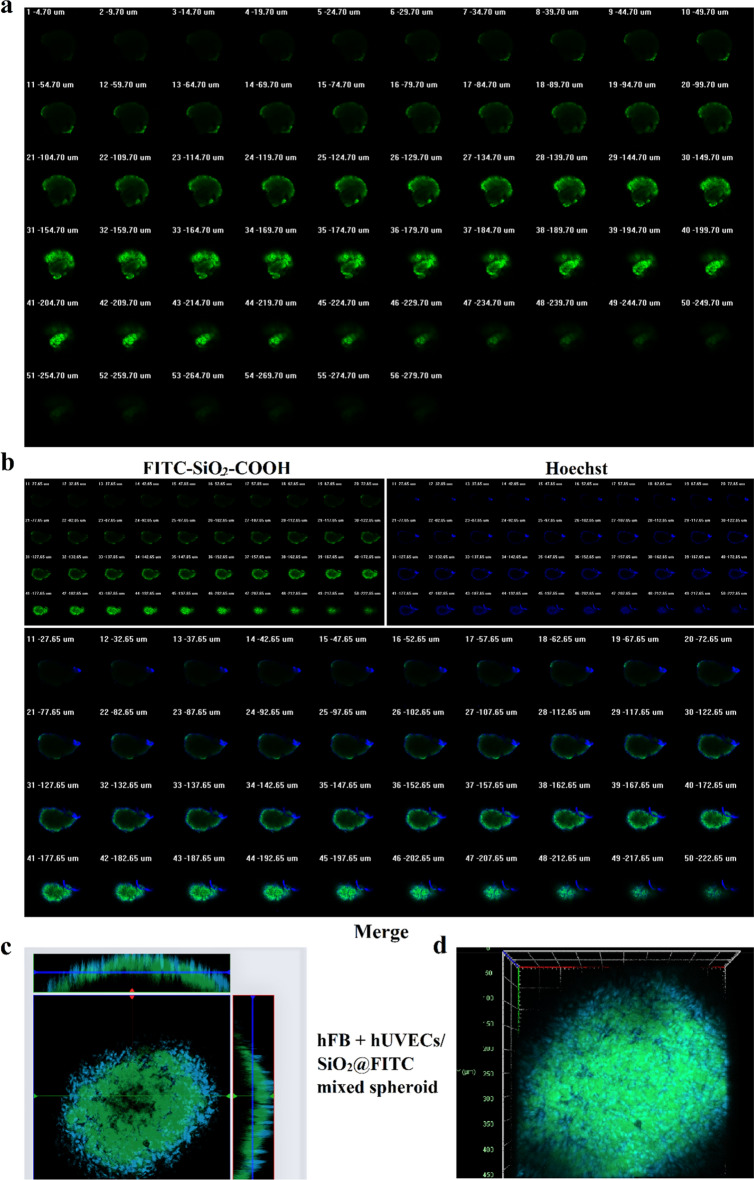
The distribution of FITC-SiO_2_ labeled cells in 3D culture. (**a**) The z-stack images of a mixture of spheroids incubated with FITC-SiO_2_-COOH NPs for 24 h. (**b**) The z-stack images of FITC-SiO_2_-COOH NPs-hFBs spheroid. The cell nuclei were visualized by Hoechst staining. The above number indicates the depth of different cell layers in the spheroid. (**c**) The mixed spheroid consisted of hFBs and FITC-SiO_2_-COOH NPs-hUVECs. Xy, yz, and xz slices of the confocal z-stack of FITC-SiO_2_-COOH (green) and Hoechst-stained cellular DNA (blue). (**d**) Confocal microscopy 3D reconstruction of the nucleus and distribution of FITC-SiO_2_-COOH NPs.

While FITC-SiO_2_-COOH NPs have established their labeling efficacy in 2D cultures, their performance in 3D cell cultures and their penetrative capabilities into deeper spheroid layers warranted investigation. In the first group, we incubated the NPs with spheroids containing both hFBs and hUVECs. After a 24-h incubation, the NPs permeated several layers (spanning layers 9–55), reaching from the apex nearly to the base of the 3D structure (Fig. 6a). In the second group, NP-incubated fibroblasts were used to create multicellular spheroids with a mean diameter of 500 µm. Results showed that the NPs emitted a strong signal across multiple cell layers even 24 h post-treatment (Fig. 6b). The FITC fluorescence mirrored the intensity of the nuclear-staining dye Hoechst, across all cellular layers (Fig. 6b). Notably, when spheroids were formulated using a mix of hFBs and NP-incubated hUVECs, a distinct cellular distribution was discerned: hUVECs populated the spheroid core while hFBs were peripherally located (Fig. 6c, d). These observations suggest that FITC-SiO_2_-COOH NPs neither inhibit cell spheroid formation nor compromise their structural integrity, validating their applicability as labeling agents in 3D cultures.

### FITC-SiO_2_-COOH NPs as a labeling agent in the Medaka fish model

**Figure 7:**
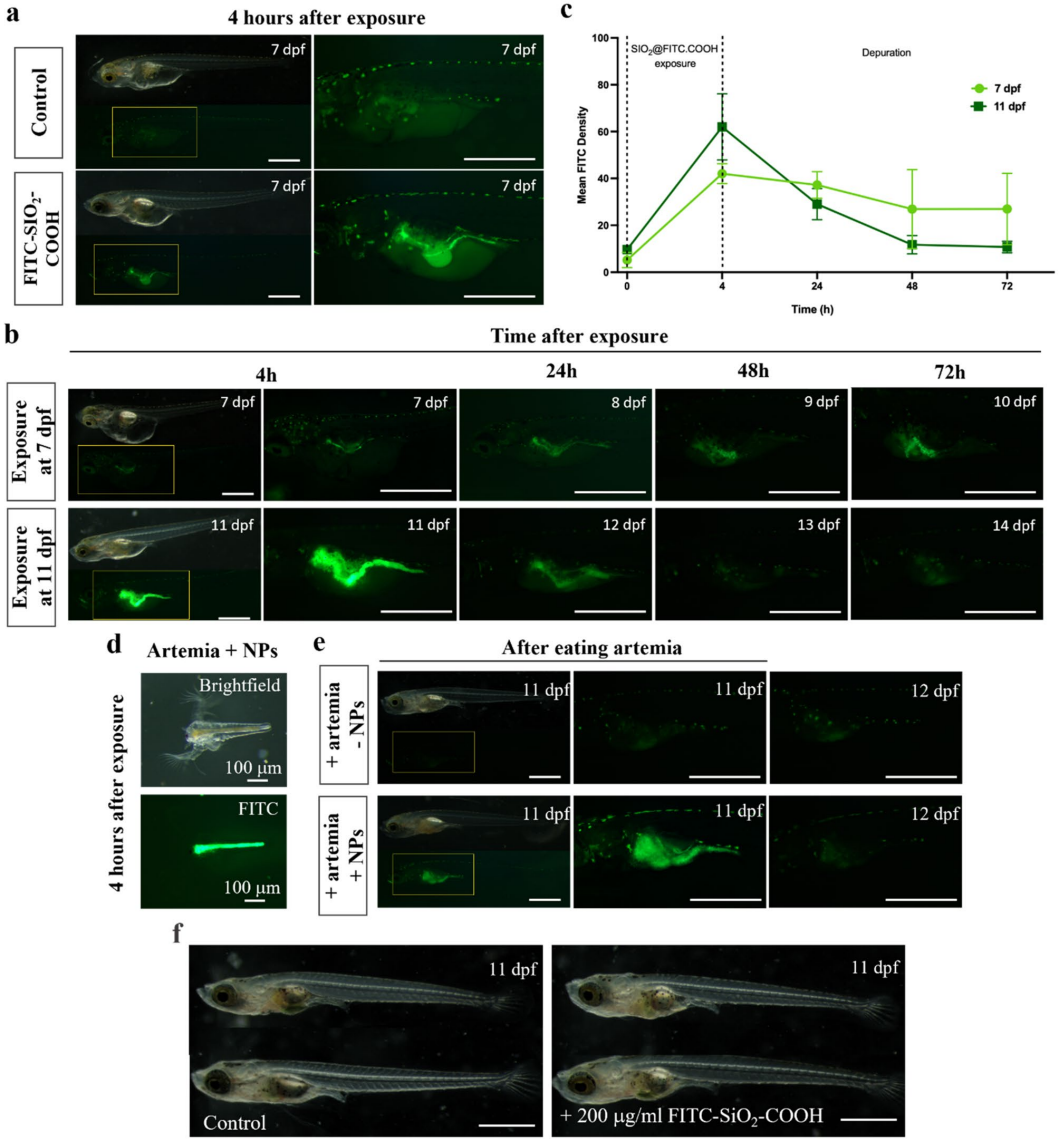
FITC-SiO_2_-COOH NPs as a labeling agent in the Medaka fish model. (**a**) The FITC signal in the small intestinal tubes of 7 dpf larvae. (**b**) The FITC signal in the small intestinal tubes of 11 dpf larvae. (**c**) Mean gray value of the FITC signal in the larvae 0, 4, 24, and 48 h after NP exposure through a 4-h feeding window. (**d**) The FITC signal in the intestinal tubes of a newly hatched live brine shrimp. (**e**) The FITC signal in the intestine of the 11-dpf fish group, which ingested the NP-ingested brine shrimp. (**f**) The morphology of the 11-dpf fishes in control and NPs treated groups.

After a 4-h exposure to a medium infused with 200 µg/ml of the NPs, FITC fluorescence was discernible in the developing gastrointestinal tract of fish larvae, a phenomenon absent in control specimens (Fig. 7a). This FITC fluorescence persisted within the small intestinal tubules of 7 dpf larvae, spreading progressively within the structure over subsequent days (spanning beyond 3 days) post-exposure. At this developmental stage, larvae predominantly rely on their yolk sac for nourishment, and minimal intestinal motility was noted (Video S1 supplemental data). Given the passive ingestion mechanisms, as larvae intake water and medium fluids, it is conceivable that the NPs gained access to the intestinal tract. These observations underscore that the NPs maintain their fluorescence for a minimum of three days in the relatively inactive intestines of the larvae. Any subsequent signal attenuation could likely be attributed to the NPs' excretion from the fish's alimentary canal rather than intrinsic fluorescence diminution. This explanation seems reasonable considering the 11 dpf larvae. Those exposed to the 200 µg/ml NP solution but deprived of feed exhibited a robust FITC fluorescence immediately after a 4-h exposure in their fully operational gastrointestinal tract (Fig. 7b) (Video S2 supplemental data). This fluorescence exhibited a marked reduction 24 h post-exposure and was virtually undetectable 48 h post-exposure (Fig. 7b, c).

Upon immersion in a medium with a concentration of 200 µg/ml for 4 h, newly hatched live brine shrimps exhibited pronounced FITC signals within their intestine. These signals persisted for several days post-exposure until the organisms succumbed (Fig. 7d). For the 11-dpf fish cohort that consumed NP-laden brine shrimp, the NP-associated fluorescence was evident in their intestines immediately after a 4-h feeding window. Yet, this fluorescence was imperceptible in the fish 24 h post-ingestion. This diminished FITC signal retention in the intestines of these fish, relative to their counterparts directly exposed to NPs, can likely be attributed to heightened digestive activity associated with brine shrimp metabolism (Fig. 7e). Beyond the gastrointestinal tract, no FITC signals were detected in vital detoxifying and excretory organs, namely the liver and kidneys, regardless of whether fish were directly exposed to NPs or consumed NP-laden brine shrimp. This suggests that the pathway for NP uptake in these fish predominantly ensues from passive water drinking or through trophic transfer; NPs likely remain unmetabolized and are subsequently expelled by the digestive system. Consequently, the retention span of NPs within the intestinal tract depends on the efficiency of the digestive system. More importantly, no developmental abnormalities or adverse or lethal effects of the NPs in the fish exposed to NPs were recorded. All tested fish remained alive and showed normal behaviors and physical activities during and for weeks following exposure (Fig. 7f).

Crucially, no developmental abnormalities or detrimental effects, lethal or otherwise, were ascribed to the NPs in the examined fish. All evaluated specimens manifested normal behavior and physiological activity both during the exposure and in the ensuing weeks.

## Discussion

Cell labeling refers to the visualization and tracking live cells over time without compromising their viability, behavior, and functionality. While NPs have been under investigation for cell tracking for roughly two decades, the surge in more innovative tracking methods has been particularly pronounced in the past 10 years^20^. Contrasting with traditional fluorescent dyes and probes, fluorescent NPs exhibit distinctive advantages of higher brightness, tunable signal, and enhanced resistance to photobleaching^21^. As alluded to in the introduction, encapsulating fluorescent NPs into a silica matrix imparts distinctive characteristics, including chemical robustness, optical transparency, biocompatibility, and facilitation of further functionalization^22^.

FITC was conjugated with APTES via coupling of the fluorescein isothiocyanate group of FITC to the amino group of the silane agent APTES. This coupling aimed to establish a covalent bond between FITC and the SiO_2_ matrix of the FITC-SiO_2_-NH_2_ NPs, which was synthesized using hydrolysis and the co-condensation reaction of tetraethyl orthosilicate (TEOS) and FITC conjugated with APTES. However, the FITC-SiO_2_-NH_2_ NPs were aggregated in the cell culture medium. This aggregation may be due to the pH value ranging from 7.6 to 7.8 in the cell culture medium, as consistent with the study of Santra et al. reporting the aggregation of FITC doped silica NPs at pH 7.4 caused by the presence of deprotonated silanol groups (Si–O_2_) on the silica surface^15^. The aggregation of NPs prevented them to efficiently penetrate the cells. Therefore, the surface of the FITC-SiO_2_-NH_2_ NPs underwent modification by integrating carboxymethylsilanetriol disodium salt, helping the NPs far from the isoelectric point. Analysis revealed that FITC-SiO_2_-COOH NPs exhibited a high negative charge with optimal dispersion in aqueous solutions. Several prior publications have reported that negatively-charged NPs have an affinity to bind to cell membranes^23–26^. This binding of negatively charged NPs to the cellular interface might arise from electrostatic interactions between the negatively charged NPs and cationic sites on the cell membrane^27,28^. Another possible mechanism to facilitate this adsorption involves an entropy-driven depletion effect^29–31^. In our study, we successfully visualized the distribution of FITC-SiO_2_-COOH NPs on the cell surface and within the cells of hUVECs and hFBs. The distinct green puncta signified the effective presence of NPs in the cytoplasm, a finding echoed in several studies that analyzed nanoparticle cellular penetration^22,32,33^.

Intriguingly, post-loading into hFBs and HeLa, the fluorescence signal persisted for up to 72 h. This signal's intensity waned to nearly half after 72 h duration compared to its level at 24 h. In the meantime, the fluorescence signal-s density in hUVECs was higher in 48 h than that in 24 h of incubation. This could be explained by the longer value of cell population doubling time in hUVECs (42 h) compared to that in hFBs (29 h) and HeLa cells (25 h). Our result was consistent with the doubling time of hUVECs, which averaged between 40 and 48h^34^. Moreover, FITC-SiO_2_-COOH NPs effectively labeled hUVECs, thereby supporting the observation of tube formation on Matrigel. Relative to commercial dyes, these NPs demonstrated a more gradual cellular penetration, yet the fluorescence signal endured significantly longer (48 h versus 16 h). This observation might hint at the engulfment mechanism of these agents by cells^35^. We noticed the commercial dye accessed the cells more rapidly, accumulating intensively within the entire cellular structure, including the nucleic^36^. However, this rapid uptake subsequently triggered cellular shrinkage and fragmentation, hallmarks of apoptosis. In contrast, the NPs generated in this study remained localized as green particles within cytoplasmic vesicles, without distorting cellular morphology. Conventionally, labeling dyes, perceived as foreign entities by cells, might trigger apoptosis if excessively internalized, especially within the nucleic. Hence, they are unsuitable for extended tracking applications. These results underscore the aptness of FITC-SiO_2_-COOH NPs for live cell labeling, with an extended capability to label 3D cultures like multicellular spheroids. After 24 h of incubation, these NPs manifested the capability to penetrate the spheroids to a depth of 234 µm. Furthermore, hFBs treated with these NPs retained their spheroid-forming ability, emitting potent fluorescence signals across all layers of the 3D structures. In the spheroids derived from a combination of hFBs and NP-labeled hUVECs, distinct cellular localizations were evident. For example, hFBs were located surrounding the outer layers, while hUVECs were found at the necrotic core. This is understandable given hFBs, with their heightened proliferative capacity compared to hUVECs, predominantly resided on the external layers, facilitating spheroid growth^37,38^. Conversely, hUVECs, known for their vascular formation capability, were predominantly situated in the nutrient and oxygen-deprived core^38^. These findings accentuate the potential of FITC-SiO_2_-COOH NPs as a viable tool for tracing living cells in both 2D and 3D cultures. Given the presence of internalized NPs within hUVECs, deeper insights into the behaviors of these cells within 3D cultures can be garnered.

For NPs to gain acceptance as live cell labeling agents, their biocompatibility is paramount; they should neither be cytotoxic nor disrupt cellular functions. The challenges and considerations associated with using fluorescent NPs in cellular contexts have been discussed in prior literature^39,40^. Thus, we evaluated the biocompatibility of FITC-SiO_2_-COOH NPs at the cellular level in this study. The data affirmed that these NPs were benign to primary cell lines, notably hFBs and hUVECs, as well as cancer cells, such as HeLa cells. Furthermore, no cellular senescence was elicited by these NPs. The morphology of the cells and cell nuclei were observably maintained without any significant change or damage. These findings are pivotal since such primary cell types rank among the most susceptible^41^. The intrinsic functionality of these cells remained unaltered in the presence of the NPs, and the quintessential migratory trait of stromal cells was preserved across cell lines. Moreover, the division of all tested cell types was also maintained in the presence of the nanoparticles. Additionally, hUVECs showcased their intrinsic angiogenic capability in vitro on Matrigel. Both hFBs and hUVECs formed spheroids in 3D cultures post-incubation with these fluorescent NPs. These collective observations validate the excellent biocompatibility of FITC-SiO_2_-COOH with stromal cells.

Our study also indicates that FITC-SiO_2_-COOH NPs manifested prolonged fluorescence signals, superior biocompatibility, and exhibited no acute in vivo toxicity in medaka fish larvae. The observation of the FITC signal in the intestine of larvae either exposed to NPs or those that consumed NP-laden brine shrimps shortly after a 4-h exposure or ingestion suggests that the primary route of NP uptake into the fish is passive ingestion through water or via their food source. As described in the results (Fig. 7), the FITC signal persisted the longest in the intestine of the 7 pdf newly hatched larvae, spanning their development for over three days post-exposure. In the 11 dpf larva exposed directly to the NP-containing medium, the signal remained visible 24 h post-exposure. However, in larvae that consumed NP-laden brine shrimps, the FITC signal was not perceptible in the intestine 24 h post-feeding. These data indicated that the NPs could retain their fluorescence signal in the intestinal tract of the fish for a minimum of 3 d following exposure, while the signal’s disappearance from the intestinal tract of the fish that consumed the NP-laden brine shrimps resulted from excretion due to digestive activity rather than instability or fading out of the signals themselves.

NPs are indigestible particles that can be excreted through the functions of the digestive system^42,43^. Larvae between 7 and 10 dpf larvae primarily rely on their yolk for sustenance^44^. Given that they are not fed and their gastrointestinal tract is in its nascent stage of development, characterized by a slender lumen and minimal intestinal motility (as observed in Video S1 supplemental data), NPs predominantly accumulate and persist within the fish intestines during this phase. By 11 dpf, although larvae can subsist on their yolk for a few additional days, they possess a functional digestive system, enabling them to begin consuming and metabolizing brine shrimp^44^. Consequently, the retention duration of NPs in the larval intestine depends on the efficacy of the digestive system. In larvae that were exposed to an NP-laden medium, the subdued digestive activity resulted in a prolonged presence of NPs and their associated signal (exceeding 24 h) within the intestinal tract. Conversely, larvae that consumed brine shrimp containing NPs exhibited heightened digestive functionality, facilitating a more rapid signal expulsion (within 24 h). This suggests that the removal of NPs from the fish’s system primarily arises from digestive excretion. Notably, the absence of the FITC signal in other parts of the fish, including vital organs like the liver and kidneys, indicates that despite their presence in the gastrointestinal tract, these NPs were not assimilated into the fish's circulatory system. This is of particular significance as the liver and kidneys are pivotal in detoxifying and expelling foreign elements that penetrate the bloodstream^44^. More crucially, the NPs exhibited no discernible acute toxic effects on the fish larvae. This assertion stems from the absence of developmental anomalies, and the lack of any detrimental or lethal outcomes in fish exposed to NPs. In other words, all tested fish remained alive and exhibited normal behaviors and physical activities, both during the exposure period and in subsequent weeks. Previous paper reported that zebrafish exposed to silica nanoparticles exhibited enduring damage to the nervous system, leading to depression, anxiety, and impaired learning and memory^45^. Additionally, pulmonary deposition of SiNPs resulted in fibrosis^46^, while Li et al.^42^ found a steady increase in infertility and sterility linked to SiNPs.

Our study demonstrated that FITC-SiO_2_-COOH NPs could be used as a passive label tracker as they could penetrate the cells and stably maintain their signal without affecting cell viability and function. Compared with the conventional dye-doped silica FITC-SiO_2_-NH_2_ NPs, our nanoparticles exhibited better dispersion in the physiological pH thanks to the surface modification by COO- group. Interestingly, this NP type could be used in different cells, including normal and cancer cells, showing the potential for broad application regarding cell types. As indicated in the results, these NPs could mark hUVECs and hFBs, two critical factors in the body's regenerative process^47^. Therefore, labeling these cells could help monitor tissue regeneration. In addition, the nanoparticles were able to label HeLa cells, suggesting that they could be used to investigate the invasion and metastasis of tumor cells and so on. Another aspect of the application is the targeting introduction of these fluorescence NPs by conjugating them with the antibodies to track specific organelles' proteins. Nonetheless, the dye-doped nanosilica synthesized by our economic method could be better for general living cell tracking.

The study had some limitations. The biocompatibility of the nanoparticle should be elucidated at the molecular range, i.e. DNA micro-damage or the intracellular transport pathways elucidating the mechanism behind the cellular uptake of FITC-SiO_2_-COOH. The in vivo testing of the NPs as a cell tracker is also important work that should be done to elucidate the application of the NPs for in vivo imaging. Even though our NPs exhibited nontoxicity to the larvae and 11-dpf fishes, more works should be performed to confirm the safety of these nanoparticles with the longer time following.

## Conclusions

The FITC-SiO_2_-COOH NPs present themselves as a potential novel fluorescence nanoparticle suited for application in biomedicine studies. Our team successfully synthesized, characterized, and functionalized the FITC-SiO_2_-COOH NPs specifically for use in live cell labeling. This nanosystem adeptly demonstrated its proficiency in delivering the fluorescence agent into hFBs, hUVECs, and HeLa cells preserving its signal integrity over extended durations in both 2D and 3D cultures. Significantly, these NPs displayed commendable biocompatibility both in vitro with the evaluated cell types and in vivo within live medaka fish larvae.

## Materials and methods

### Animal care

The biocompatibility and potential toxicity of FITC-SiO_2_-COOH nanoparticles were investigated in vivo using wild-type medaka fish (*Oryzias latipes*) larvae as a model system. Larvae at both 7 days post-fertilization (dpf), having a body length of approximately 5 mm, and 11 dpf were selected for the studies. The 7 dpf larvae, being newly hatched, possess a rudimentary gastrointestinal tract with limited activity. At this stage, nutrients are primarily sourced from the yolk sac, obviating the need for external food consumption for several additional days. By 11 dpf, the larvae exhibit a more developed gastrointestinal tract^43^ and, while still utilizing yolk nutrients, begin to ingest external food. The experiments aimed to ascertain whether digestive activity influenced the residence time of NPs within the intestinal tract of fish at this developmental stage.

### Ethical statement

All experiments complied with the national and institutional laws and are reported in accordance with the ARRIVE guiedlines. All fish care and experimental protocols adhered to established methodologies^43^ and were approved by the Dinh Tien Hoang Institute of Medicine, Hanoi, Vietnam (Approval number: IRB-AR.002).

### Chemicals and materials

Fluorescein isothiocyanate isomer I (FITC, ≥ 90%), tetraethyl orthosilicate (TEOS, 98%), (3-Aminopropyl) triethoxysilane (APTES, 98%), and ammonia hydroxide (NH_4_OH, 28%) were obtained from Sigma Aldrich. Ethanol, HCl, and NaOH were purchased from Merck. Carboxymethylsilanetriol disodium salt, 25% (CETSS, SIC 2263) was provided by Gelest Inc. DI water was used for all experiments. All reagents were used directly without further purification.

### Instrumentation

TEM measurements were performed on a JEOL 1400 flash Jeol microscope operating in the 120 kV voltage range. SEM measurements were performed on a Hitachi S-4800 operating in the 5 to 10 kV voltage range. The samples for the analysis were prepared by dropping a sample solution on the silicon wafer and drying at room temperature. DLS (Nano ZS. Malvern) was used to determine the size distribution and zeta potential values. A Spectrum Two FT-IR spectrometer, Perkin Elmer, and a Labram HR evolution Raman Spectrometer, Horiba, were used to record the FTIR and Raman spectra, respectively. A Shimadzu UV2600 spectrophotometer measured UV–Vis absorption. The fluorescent spectra were measured on a Cary Eclipse fluorescence spectrophotometer.

### Preparation for FITC-SiO_2_-NH_2_

To synthesize FITC-conjugated APTES molecules, 3 mg FITC, 6 µL APTES, and 60 µl KOH 1 M were combined in 1 mL of an ethanol solution. This mixture was kept in the dark and stirred overnight. Following this, FITC-SiO_2_-NH_2_ NPs were fabricated using a modified Stober method. Specifically, 1 mL of TEOS and 1 mL of the previously prepared FITC-conjugated APTES were incorporated into a solution containing 100 mL ethanol and 6 mL NH_4_OH. This mixture was then stirred vigorously at ambient temperature for 24 h. The resultant FITC-SiO_2_-NH_2_ NPs were isolated via centrifugation, rinsed with ethanol thrice, and finally resuspended in deionized (DI) water.

### Surface modification of Fluorescent Dye-Doped silica NPs (FITC-SiO_2_-COOH)

The FITC-SiO_2_-NH_2_ sample was functionalized by carboxyethysilanetriol disodium, an organosilane bearing a carboxyl functional group. In a standard procedure, approximately 8 µL of carboxyethysilanetriol disodium was introduced into 10 mL of FITC-SiO_2_-NH_2_ solution under sonication for 30 min. Then, 10 µL of NH_4_OH was added, and the mixture was continuously stirred for 6 h. The resulting FITC-SiO_2_-COOH particles were washed several times with DI water by centrifugation and dispersed in DI water for subsequent use.

To evaluate the simultaneous influence of pH, the ability to resist chemicals, especially salt at high concentration, on the colloidal properties of nanoparticles, we introduced FITC-SiO2-COOH NPs into a solution of citric/citrate buffer systems and NaHCO_3_ buffer system to adjust pH from 4 to 9. The total concentration of substances participating in creating the buffer system was 100 mM. Then, the solutions with the same nanoparticle concentration in the different pH buffer solutions prepared above were evaluated for the system's colloidal stability by examining the zeta potential, the hydrodynamic diameter of nanoparticles, and their poly index dispersion values.

### Cell culture

Primary cells isolated from human umbilical cord tissue, namely fibroblasts (hFBs) and umbilical vein endothelial cells (hUVECs), provided by the project VINIF.2020.DA07. hFBs, were cultured in DMEM/F12 medium (Gibco, USA) supplemented with 10% fetal bovine serum (FBS, Gibco, USA), 100 units/mL of penicillin, and 100 µg/mL of streptomycin (Gibco, USA). The hUVECs were cultured in an EBM-2 medium kit (Lonza, Swiss). HeLa cells were gifted from Professor Stefan Dimitrov at Institute Albert Bonniot (present name is Institute for Advanced Biosciences). These cells were cultured in DMEM (Gibco, USA) supplemented with 10% fetal bovine serum (Gibco, USA). All the cells were cultured at 37 °C with 5% CO_2_.

### Cell viability assay

The potential cytotoxic effects of the FITC-SiO_2_-COOH NPs on various cell types were evaluated using the MTT assay, adhering to the manufacturer's protocol (Promega, Madison, WI, USA). Cells were seeded onto 96-well plates at a density of 2.5 × 10^2^ cells per well and subsequently incubated at 37 °C for 24 h in a humidified atmosphere with 5% CO_2_. The cells were then exposed to nanoparticle concentrations of 1, 10, 50, and 100 μg/mL and further incubated for 24, 48, and 72 h. Post-incubation, 15 µL of the MTT labeling reagent dye solution (with a final concentration of 0.5 mg/mL) was added to each well. Following incubation and solubilization steps, cellular absorbance was measured at 570 nm using an Elisa plate reader (BioTech Power Wave XS, Winooski, VT, USA). Each experiment was performed in triplicate.

### Wound healing assay

Cells were cultured in 24-well plates at a density of 8 × 10^3^ cells/cm^2^. Upon reaching a confluency greater than 95%, cells were treated with mitomycin (10 µg/mL) for 2 h to inhibit cell proliferation. Subsequent to this treatment, a scratch was introduced using a scratcher (SLP, Korea). The cells were then treated with FITC-SiO_2_-COOH nanoparticles at concentrations of 50 and 100 µg/mL. The wound healing progression was monitored and documented using an optical microscope. Captured images were subsequently analyzed using ImageJ software (version 1.46r).

### Senescence cell analysis

The proportions of senescent cells in both hUVECs and hFBs were determined using the Senescence Cells Histochemical Staining Kit (Sigma-Aldrich, Missouri, USA). Cells were seeded in 6-well plates at a density of 2 × 10^4^ cells/cm^2^ and incubated with FITC-SiO_2_-COOH NPs at concentrations of 50 μg/mL or 100 μg/mL at 37 °C and 5% CO_2_ for 48 h. Post incubation, the culture medium was aspirated and cells were briefly rinsed with phosphate-buffered saline (PBS) prior to fixation with 1X Fixation buffer for 7 min. Following two PBS washes, cells were treated with the staining mixture and incubated overnight at 37 °C in a 5% CO_2_ environment. After discarding the staining mix and rinsing with PBS, cells underwent DAPI staining (Abcam, Cambridge, UK) for 15 min. The samples were visualized and documented using both optical and fluorescence microscopes (Olympus, Tokyo, Japan), with the resultant images analyzed using ImageJ software (version 1.46r).

### The angiogenesis assay

hUVECs, cultured in EBM-2 medium until 80% confluency was attained, were utilized for angiogenesis testing with the Angiogenesis Assay Kit (Abcam, UK), following the manufacturer's protocol. Cells were seeded into 6-well plates, achieving a density of 2 × 10^5^ cells/well. Each well was pre-coated with extracellular matrix solution. Experimental groups were treated with 100 μg/mL of FITC-SiO_2_-COOH NPs in hUVECs. Observations of the angiogenesis process were documented using fluorescence microscopy at the 490/540 nm wavelength, and subsequent image analysis was conducted with Image J software (version 1.46r).

### 2D cell labeling

Cells were seeded onto glass coverslips placed within 24-well plates and incubated for 24 h in appropriate media. Following incubation with varying concentrations of FITC-SiO_2_-COOH NPs, cells were rinsed, fixed with a solution containing 4% paraformaldehyde and 2% sucrose at 37 °C for 15 min, permeabilized with 0.2% Triton X-100 for 10 min, and blocked using 5 mg/mL BSA. Cells were further incubated with mouse monoclonal anti-human α-tubulin antibodies or anti-human CD63 antibodies for 1 h, followed by a 30-min incubation with Alexa 546-conjugated anti-mouse antibodies. Nuclei were stained with Hoechst 33342. Images were acquired using a ZEISS 510 Laser Scanning Confocal microscope equipped with either 40× or 63× objectives, with subsequent analysis performed using the LSM imaging browser software.

### Fluorescence intensity measurement

All images were acquired with Apotome II Microscope (Zeiss, Germany). The fluorescent images were then analyzed using ImageJ software (version 1.48) by selecting one cell at a time in an image and measuring the area, integrated density, and mean gray value. Using the calculation for corrected total cell fluorescence (CTCF) = integrated density–(area of selected cell × mean fluorescence of background readings), as described by McCloy et al.^48^, the fluorescence intensity of each cell was calculated using Excel (Microsoft Office 365) and GraphPad Prism (version 8.4.3; GraphPad Software). For each image, three background areas were used to normalize against autofluorescence. Each biological sample was grown at different time points, and for each condition, 3 images were acquired with a 40× objective, which was then used for statistical analyses.

### 3D cell computed chromatography

Multicellular spheroids were generated using a modified hanging drop method, as previously described^21^. Briefly, 15 µl of medium containing 5 × 10^3^ cells were applied to each circle on an inverted cover and plated on a 96-well plate to form individual spheroids. The inverted cover was then positioned onto an agarose-coated plate. Following 48 h of incubation, spheroids were transferred to agarose-coated wells for extended cultivation. Spheroids were exposed to the nanoparticles in one of two manners: (1) addition of NPs post spheroid transfer with a 24-h incubation, or (2) a 24-h pre-incubation of cells with NPs prior to spheroid formation. The cell nuclei of the spheroids were stained using Hoechst 33342. Morphological and fluorescence observations of the spheroids were captured using a Nikon Eclipse Ti microscope.

### In vivo assessment of FITC-SiO_2_-COOH NPs on Medaka model

For the experiments, individual larvae were housed in 24-well cell culture plates, each containing 0.5 ml of E3 rearing medium, with or without the nanoparticles. Larvae at 7 dpf (n = 5) were exposed to FITC-SiO_2_-COOH NPs by adding the particles to the rearing medium to achieve a concentration of 200 µg/ml. After a 4-h immersion in this medium, larvae were transferred back to the standard E3 rearing medium. Control larvae (n = 5) underwent a similar protocol, sans nanoparticle exposure. Fluorescent images of the fish were captured before, immediately post the 4-h nanoparticle exposure, and daily thereafter to monitor the FITC signal from the nanoparticles.

For the 11-dpf larvae, three experimental groups were established: (1) Larvae directly exposed to a medium containing 200 µg/ml of nanoparticles (n = 5); (2) Larvae fed with live brine shrimp previously ingested with nanoparticles (n = 5); (3) Control group: Larvae fed with regular, untreated brine shrimp. The experimental procedure for the 11-dpf fish paralleled that of the 7 dpf cohort. However, for the brine shrimp-fed groups, larvae were maintained in E3 medium and provided with either brine shrimp exposed to 200 µg/ml nanoparticles or regular brine shrimp over a 4-h period.

All fish were housed at temperatures ranging from 28 to 30 °C within recirculating systems and subjected to a controlled light cycle (14 h light, 10 h dark). Both embryos and larvae were raised in E3 medium (comprising 5 mM NaCl, 0.17 mM KCl, 0.33 mM CaCl_2_, and 0.33 mM MgCl_2_) at 30 °C, with daily medium replacements. Each experimental and control group consisted of five embryos or larvae.

## Supplementary Information

Supplementary Information 1.

Supplementary Video 1.

Supplementary Video 2.

## Data Availability

The data sets generated during and/or analyzed during the current study are available from the corresponding author on reasonable request.

## Abbreviations

FITC: Fluorescein isothiocyanate isomer
NPs: Nanoparticles
APTES: (3-Aminopropyl) triethoxysilane
SEM: Scanning electron microscopy
DLS: Dynamic light scattering
TEOS: Tetra ethyl orthosilicate
DI: Water Deionized Water
HD: Hydrodynamic diameter
hUVECs: Human umbilical vein endothelial cells
hFBs: Fibroblasts
DMEM/F12: Dul Dulbecco's Modified Eagle Medium/Nutrient Mixture F-12
FBS: Fetal bovine serum
EBM-2: Medium endothelial Cell Basal Medium EBM 1
PBS: Phosphate buffer saline
PdI: Polydisperse Index
