# Author Correction: High biocompatible FITC-conjugated silica nanoparticles for cell labeling in both in vitro and in vivo models

**DOI:** 10.1038/s41598-025-10067-1

**Published:** 2025-07-11

**Authors:** Thi Thuy Nguyen, Hoang Nam Nguyen, Thi Ha Lien Nghiem, Xuan-Hai Do, Thanh Thuy To, Thi Xuan Phuong Do, Dieu Linh Do, Huong Giang Nguyen, Huy Manh Nguyen, Ngoc Dinh Nguyen, Manh Quynh Luu, Trong Nghia Nguyen, Thi Bich Ngoc Nguyen, Van Toan Nguyen, Van Thanh Pham, Uyen Thi Trang Than, Thi My Nhung Hoang

**Affiliations:** 1 Center for Quantum and Electronics, Institute of Physics, Vietnam Academy of Science and Technology, 18 Hoang Quoc Viet Street, Hanoi, Vietnam; 2 Nano and Energy Center, VNU University of Science, Hanoi, 334 Nguyen Trai Street, Thanh Xuan, Hanoi, Vietnam; 3 Department of Practical and Experimental Surgery, Vietnam Military Medical University, 160 Phung Hung Street, Phuc La, Ha Dong, Hanoi, Vietnam; 4 Faculty of Biology, VNU University of Science, Hanoi, 334 Nguyen Trai Street, Thanh Xuan, Hanoi, 10000 Vietnam; 5 Faculty of Physics, VNU University of Science, Hanoi, 334 Nguyen Trai Street, Thanh Xuan, Hanoi, Vietnam; 6 Vinmec Hi-Tech Center and Vinmec-VinUni Institute of Immunology, Vinmec Healthcare System, 458 Minh Khai Street, Hanoi, Vietnam

Correction to: *Scientific Reports* 10.1038/s41598-024-55600-w, published online 23 March 2024

The original version of this Article contained an error in Figure 3, where in panel (C), the 50 ug/mL-treated one is the duplicate of the control.

**Fig. 3:**
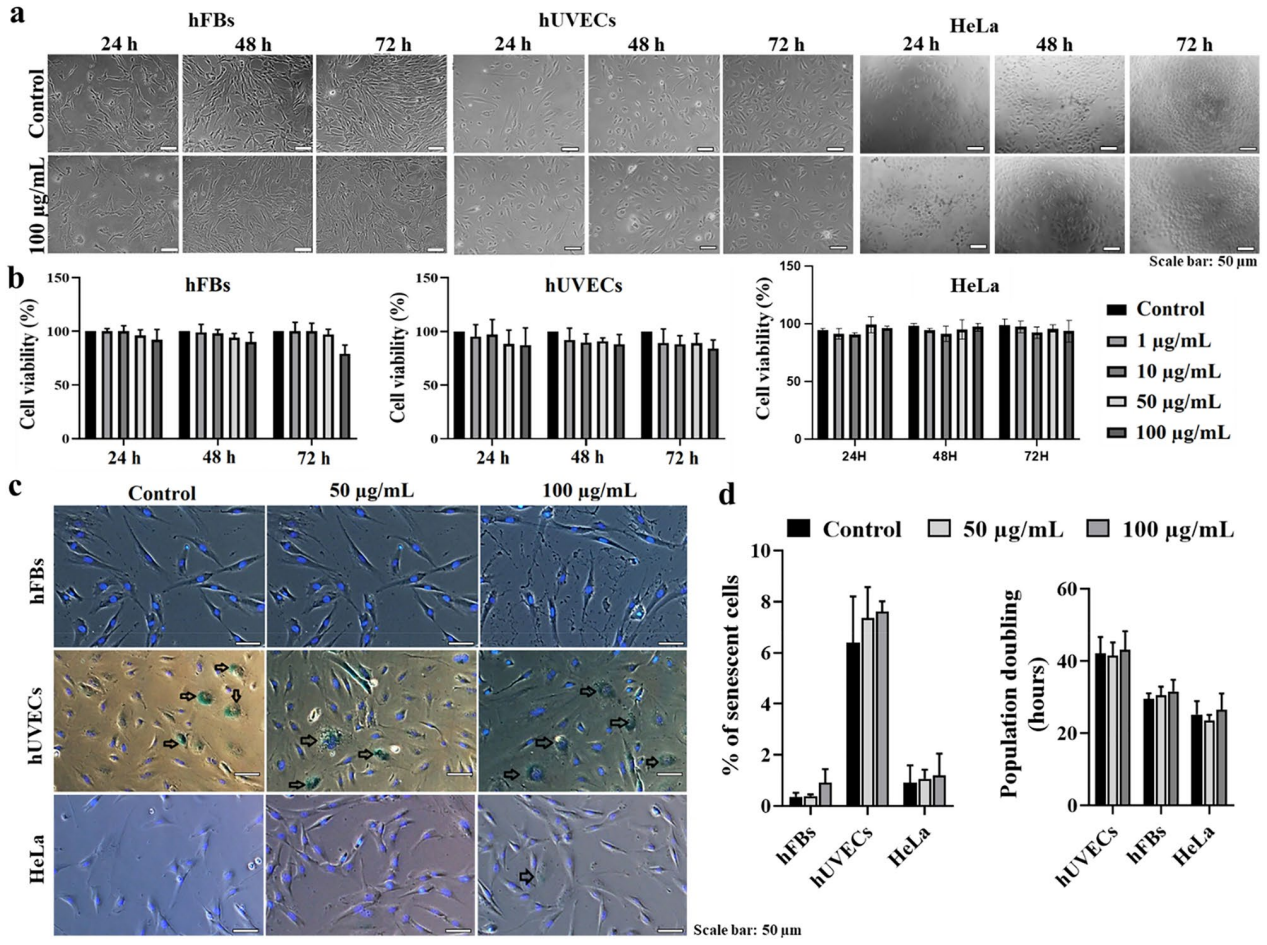
The effect of FTIC-SiO_2_-COOH NPs on cytotoxicity and cell senescence. (**a**) The morphology and cell density of hFBs, hUVECs, and HeLa cells in the presence of the NPs. (**b**) The cell viability (%) of hFBs, hUVECs, and HeLa cells with different doses assessed at 24, 48, and 72 h. (**c**) The cellular senescence signals detected by β-galactosidase staining; black arrows indicate aging cells. Cell nuclei were stained with Hoechst (blue). (**d**) The percentage of senescence cells (%) and the population doubling time among hFBs, hUVECs, and HeLa cells***.*** Data was collected from three biological trials (n = 3) and presented as mean ± SD.

The original Article has been corrected.

