# Supplementary material for: High biocompatible FITC-conjugated silica nanoparticles for cell labeling in both in vitro and in vivo models
Source: Sci Rep. 2024 Mar 23;14:6969. doi: 10.1038/s41598-024-55600-w (PMC10960792; doi:10.1038/s41598-024-55600-w)
Supplement: Supplementary file 1 — Supplementary Information 1. [file 41598_2024_55600_MOESM1_ESM.docx]

**Description of Additional Supplementary Files**

**Supplementary Movie 1: FITC fluorescence in 7 dpf larvae.** The signal of FITC-SiO_2_-COOH NPs persisted within the small intestinal tubules. At this developmental stage, larvae predominantly rely on their yolk sac for nourishment, and minimal intestinal motility was noted.

**Supplementary Movie 2: FITC fluorescence in 11 dpf larvae.** Those exposed to the 200 µg/ml NP solution but deprived of feed exhibited a robust FITC fluorescence immediately after a 4-h exposure in their fully operational gastrointestinal tract.
